# Continuation rates of alpha-blockers mono-therapy in adult men, prescribed by urologists or general practitioners: a pharmacy-based study

**DOI:** 10.1007/s00345-018-2557-3

**Published:** 2018-11-13

**Authors:** Ilse M. J. Hordijk, Martijn G. Steffens, Eelko Hak, Marco H. Blanker

**Affiliations:** 1 Department of General Practice and Elderly Medicine, University of Groningen, University Medical Center Groningen, HPC FA21, PO Box 196, 9700 AD Groningen, The Netherlands; 2 Department of Urology, Isala Clinics Zwolle, Zwolle, The Netherlands; 3 Faculty of Science and Engineering, Groningen Research Institute of Pharmacy, University of Groningen, Groningen, The Netherlands

**Keywords:** α-Blockers, Prescription patterns, Urologists, General practitioners, Lower urinary tract symptoms

## Abstract

**Purpose:**

α-Blockers are commonly used for the treatment of male lower urinary tract symptoms (LUTS). The Dutch GP guideline on male LUTS contains an advice to discontinue treatment after 3–6 months of successful treatment. The guideline for urologists does not support this advice. It is unclear if these differences lead to other patterns of (dis)continuation of α-blockers. We aim to study continuation rates of α-blockers, prescribed by a urologist or a general practitioner (GP), and to predict discontinuation after 1 year.

**Methods:**

We conducted a retrospective inception cohort study on prescription patterns of α-blockers among Dutch men between 2006 and 2014, using the IADB.nl pharmacy prescription database from the University of Groningen. We selected men aged 30 years or older with a first α-blocker prescription between 2006 and 2013, and analysed continuation of prescriptions.

**Results:**

The database included 12,191 individual patients with at least one α-blocker prescriptions from a urologist (44.5%) or a GP (55.5%). The median treatment period for patients who started in the GPs office was 210 days, compared to 150 days for patients with a prescription from a urologist. Of all patients, 60.3% (GP prescriptions) and 66.1% (urologists’ prescriptions) had discontinued treatment (Chi-square *p* < 0.001). Discontinuation rates were age dependent with higher rates in the youngest age groups.

**Conclusion:**

In this study, the discontinuation rate 1 year after the initiation of treatment was high. Although Dutch GP’s and urologist’s guidelines differ with respect to a discontinuation advice, we could not find clinically relevant difference in (temporary) discontinuation rates.

**Electronic supplementary material:**

The online version of this article (10.1007/s00345-018-2557-3) contains supplementary material, which is available to authorized users.

## Introduction

The pharmacological treatment in male LUTS generally starts with α-blocker therapy [1–3]. The Dutch general practitioner (GP) guideline “male LUTS” contains an advice to discontinue α-blocker therapy after 3–6 months of use [3]. This advice is based on the overall variable LUTS severity over time [4], but has no further evidence base. The guideline of Dutch urologists does not support this recommendation; neither does the guideline of the European Association of Urologists (EAU) [2]. In the latter, discontinuation of α-blockers is only suggested for men who receive combination treatment with a 5-α-reductase inhibitor [2].

Whether this difference in guideline advises leads to other prescription patterns between GPs and urologists has not been studied. We evaluated prescription patterns in the first year of α-blocker treatment in men initiated by Dutch GPs and urologists. We examined discontinuation rates as well as predictors for discontinuation in incident patients on α-blockers.

## Materials and methods

### Study design, data source and study population

We have conducted a retrospective inception cohort study. In this study, we analysed prescription patterns of α-blockers between 2006 and 2014 in Dutch men. The University of Groningen IADB.nl pharmacy prescription database (IADB.nl) provided all data. The IADB database contains information on all prescriptions dispensed by 60 Dutch community pharmacies [5]. This includes over 1.2 million patients with more than 100 million unique prescriptions. The IADB database includes no information on the indication for treatment. Nor does it contain additional patient information, other than age and sex. Population distribution and drug use were to a large extent comparable to the Dutch general population [5].

We selected men aged 30 years or older who had a first α-blocker mono-therapy prescription between 2006 and 2013, based on the anatomic therapeutic chemical classification (ATC)-codes G04CA01, G04CA02, G04CA03 and G04CA04. These codes do not include α-blockers used for the treatment of hypertension. We did not select new prescriptions of combination therapy (ATC codes G04CB, G04CA52, and G04CA53); as such, therapy is not recommended in the Dutch GP guideline [3] and our main aim was to compare prescriptions from GPs with prescriptions from urologists.

A prescription was considered a first prescription if there were no identical prescriptions present in the two preceding years. We categorised the first prescriber as either GP or specialist/urologist. For every included patient who could be followed for 52 weeks, we retrieved all dispensed α-blocker prescriptions and other urologic prescriptions from the database. Within the IADB database, patient status is listed as active or inactive, due to moving out of the area or dying. In the Netherlands, the vast majority of patients obtained their medications from one pharmacy, mostly situated nearby the patients’ home.

We calculated the total duration of α-blocker use in days, by dividing the number of doses dispensed by the pharmacy by the number of doses per day and evaluated drug usage at 2, 6, 13, 26, 39 and 52 weeks after the date of first prescription. We classified patients classified as “discontinued” if the total duration (in days) was less than these periods. If total duration exceeded these periods, we classified patients as “current users”. We did this for each point in time. For “current users”, we examined if at any time interval, the patient had had a period of discontinuation. We defined a period of discontinuation if 15 tablets or more were missing in the preceding period. So this reflects a gap in refills. With this information, we classified current users as “temporary discontinuation” or “no temporary discontinuation”.

### Statistical analysis

We present descriptive statistics with mean ± standard deviation for continuous data or percentage for categorical data. We compared the differences in (temporary) discontinuations rates between GPs and urologists, age groups (30–49, 50–59, 60–69, 70–79 and 80–100 years), and year of first prescription, using Chi-square tests. Next, we entered these possible predictors in a multivariable logistic regression analysis to predict current use after 1 year. We calculated the odds-ratio and corresponding 95% confidence interval (95% CI). We applied the Nagelkerke *R*^2^ to assess the percentage of explained variance and the Hosmer–Lemeshow test to determine the goodness-of-fit of our model. We considered a *p* value < 0.05 as statistically significant. All analyses were performed using SPSS Version 25.

## Results

We identified 12,191 individual patients with one or more α-blocker prescriptions. In 5419 cases, urologists provided the first prescription. In the other 6772 cases (55.5%), GPs were the prescribers. Baseline characteristics are presented in Table 1.

**Table 1:** Baseline characteristics of included patients

Characteristics	All	First prescription by general practitioner	First prescription by urologist
*N* (%)	12,191	6772 (55.5)	5419 (44.5)
Age, mean ± SD	65.3 ± 12.2	65.8 ± 11.8	64.6 ± 12.7
Age group, *N* (%)			
30–49	1281 (10.5)	554 (8.2)	727 (13.4)
50–59	2483 (20.4)	1436 (21.2)	1047 (19.3)
60–69	3836 (31.5)	2219 (32.8)	1617 (42.2)
70–79	3004 (24.6)	1645 (24.3)	1359 (25.1)
80–100	1587 (13.0)	918 (13.6)	669 (12.3)
α-Blocker, *N* (%)			
Alfuzosin	3175 (26.0)	2227 (32.9)	948 (17.5)
Tamsulosin	8981 (73.7)	4535 (67.0)	4445 (82.0)
Terazosin	8 (0.1)	5 (0.1)	3 (0.1)
Silodosin	27 (0.2)	5 (0.1)	22 (0.4)
Combination therapy, *n* (%)	935 (7.7)	241 (3.6)	694 (12.8)
Median duration of therapy, days (IQR)	180 (45–857)	210 (45–735)	150 (45–735)

**Fig. 1:**
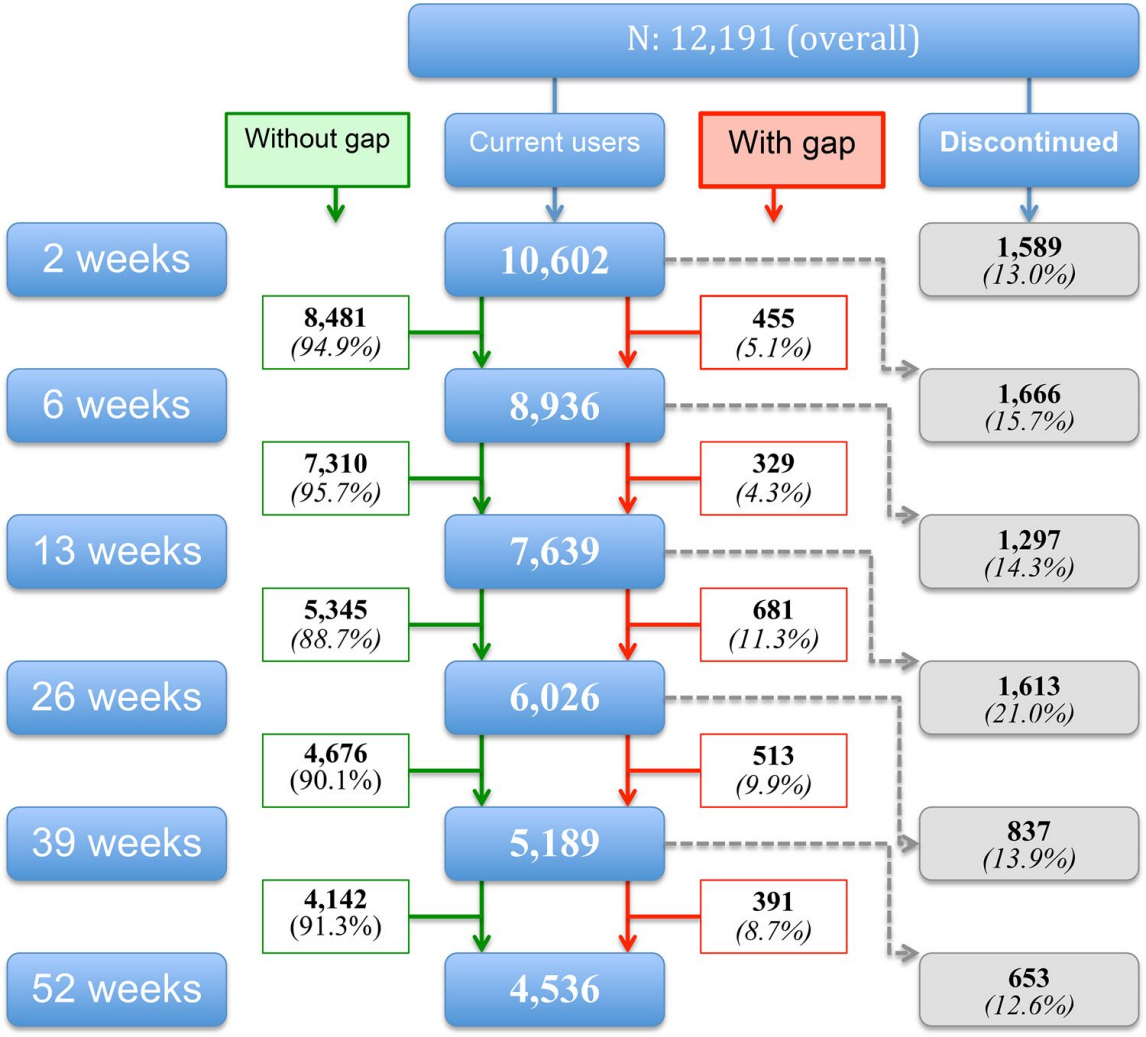
Numbers and percentages of patients who discontinued and continued α-blocker treatment (with or without gap)

The median treatment period for patients who started in the GPs office and in hospital was 210 days and 150 days, respectively. Figure 1 shows the number of current users at 2, 6, 13, 26 and 52 weeks for the complete sample. These numbers showed no relevant differences between the two prescriber groups (see supplementary file). Of all patients, 60.3% and 66.1% of, respectively, GP’s and urologists’ patients had discontinued treatment (Chi-square *p* < 0.001) within 1 year after initiating α-blocker treatment.

Discontinuation rates were age dependent with higher rates in the youngest age groups (see Table 2). At each point in time, we found the differences to be statistically significant (all Chi-square, *p* < 0.05).

**Table 2:** Cumulative percentage of complete discontinuation according to age and time

Time (weeks)	30–49 years	50–59 years	60–69 years	70–79 years	> 80 years
0–2	27.8%	15.1%	10.3%	9.3%	11.5%
2–6	50.9%	29.0%	22.6%	21.8%	22.8%
6–13	65.2%	41.1%	32.7%	31.7%	31.9%
13–26	78.7%	56.1%	46.1%	42.4%	45.3%
26–39	83.5%	62.5%	53.7%	49.6%	52.4%
39–52	86.7%	68.3%	58.7%	55.9%	58.0%

The differences between age categories were statistically significant at each point in time (Chi-square, *p* < 0.05)

Age, prescriber and year of first prescription were independent predictors of current use after 1 year of treatment in the multivariable logistic regression analysis (see Table 3). The percentage of explained variance for this model was 6.1% (Nagelkerke *R*^2^).

**Table 3:** Predictors of current use 1 year after initiation of α-blocker therapy

Factors	Odds ratio (95% CI)
Prescriber	
General practitioner	1.00 (referent)
Urologist	0.81 (0.75–0.87)
Age	
30–49	1.00 (referent)
50–59	2.91 (2.42–3.49)
60–69	4.45 (3.74–5.29)
70–79	5.01 (4.19–5.97)
80–100	4.56 (3.77–5.51)
Year of first prescription	
2006 and 2007	1.00 (referent)
2008 and 2009	0.97 (0.87–1.08)
2010 and 2011	0.93 (0.04–1.03)
2012 and 2013	0.78 (0.70–0.86)

Hosmer–Lemeshow = 0.24, Nagelkerke *R*^2^ = 0.061

## Discussion

This pharmacy-based study shows that the majority of men who start an α-blocker discontinue its use within 1 year. In this respect, no clear differences are shown between patients with a first prescription from a GP or from a urologist. Only a minority of men who still use the α-blocker after 1 year have had a period of discontinuation.

The main limitation of this database study is that no clinical information is available from the patients and indications for prescriptions are not recorded which might have led to misclassification. Information was limited to patient age, date of prescription, and prescriber. In the Netherlands, α-blockers are also prescribed for stone expulsive therapy and in case of acute urinary retention to support trial without catheter [3]. The incidence of both problems is generally low, although especially urolithiasis might explain the high discontinuation rate after 6 weeks (50.9%) in the youngest age group.

Next, it is unclear if patients discontinued α-blocker use themselves, for example due to side effects or a lack of effectiveness, or if physicians actively gave the advice to stop its use. We expect that short-term use may especially be associated with lack of effect on symptom severity, side effects, or with other indications, such as urolithiasis. Previously, Koh et al. showed that resolved symptoms (31.1%), no symptom improvement (23.7%) and adverse events (20.0%) were the main reasons for discontinuation [6]. Another limitation may be the low percentage of explained variance of the regression model. This shows that other variables contribute to the treatment patterns, which were not taken into account in the analyses. In the American cohort study, the percentage of explained variance was not reported [7].

The main strength of this study is the large number of patients from whom information is available. This database is representative for the Netherlands [5]. Most patients in the Netherlands collect their prescriptions from a single pharmacist. α-Blockers are by no means available without a prescription. In the Netherlands, the pharmacists dispense each first prescription only for 2 weeks with an automated extension of this period based on the prescription. If patients did not understand that usage was meant for a longer period, this could explain part of the discontinuation after 2 weeks.

Although α-blockers are generally considered to be effective for the treatment of male LUTS, the discontinuation rate was high in this study, as well as in three other studies [6–8]. In the first study, it was is unclear who was the first prescriber [8], whereas in the second study a combination of α-blocker and 5-α-reductase inhibitor prescriptions from a urologist was examined [6]. More recently, Rensing et al. showed in a large American cohort that discontinuation most frequently occurred in the first year after initiating treatment and resulted in a cumulative incidence of 53.1 (urologists) and 55.4% (PCPs) after 3 years [7]. For all prescriptions (α-blockers, 5ARI and anticholinergics), authors found a statistically significant difference in discontinuation rates between urologists and PCPs: hazard ratio 1.19 (CI 95% 1.09−1.29) [7]. We feel that this small difference is not clinically relevant.

A possible explanation of the high discontinuation in these and our studies may be that many men do not experience a clinically relevant effect, because LUTS have a multifactorial origin whilst α-blockers only influence one specific aspect [9, 10]. Although this is recognised in current guidelines, especially due to the low number of side effects and low costs of α-blockers, these drugs are advised for all men with uncomplicated LUTS who opt for active treatment.

Shortridge et al. showed much lower discontinuation rates, in a study using electronic medical records of 1807 patients with LUTS/BPH: 11.6% after 1 year [11]. This large difference may be explained by the inclusion criteria applied. To meet the inclusion criteria in that study, participants were required to have two or more prescriptions. So the high discontinuation rates in the first weeks of use, as shown in our study population, are excluded in that study. Finally, in the largest database on this topic, Cindolo et al. showed that the 1-year adherence in Italian men exposed to at least 6 months of mono-therapy or combination therapy [12]. The authors found that only 29% of these men adhered to therapy. We cannot explain the very large difference with our results. In men who used α-blockers for at least 6 months, 75% still used their medication after 1 year.

α-Blockers are the mainstay of pharmacological treatment of male LUTS. This is a “one size fits all” or “trial and error” strategy, focussing on one aspect of the male lower urinary tract. Defining subgroups of men with a higher chance of successful α-blocker treatment would serve patients and their physicians, and reduce the high discontinuation rates, simply by not initiating this treatment if the chance of success is low. As it is unlikely that new randomised controlled trials will be conducted for this drug group, observational studies based on real life practice may provide support for a more personalised approach.

## Conclusion

We found a high discontinuation rate after 1 year of treatment, with about two-thirds of new patients discontinued treatment within 1 year. A period of discontinuation was seen in only 30% of patients who still used α-blockers 1 year after treatment initiation. Despite the differences between Dutch GP’s and urologist’s guidelines with respect to a discontinuation advice, the differences in (temporary) discontinuation rates described in this study are not clinically relevant.

## Electronic supplementary material

Below is the link to the electronic supplementary material.

Supplementary material 1 (PDF 446 kb)

Supplementary material 2 (PDF 443 kb)
