# Supplementary material for: Continuation rates of alpha-blockers mono-therapy in adult men, prescribed by urologists or general practitioners: a pharmacy-based study
Source: World J Urol. 2018 Nov 13;37(8):1659–64. doi: 10.1007/s00345-018-2557-3 (PMC6684751; doi:10.1007/s00345-018-2557-3)

Figure 2. Numbers and percentages of patients who discontinued and continued α-blocker treatment (with or without gap) with first prescription by general practitioner.

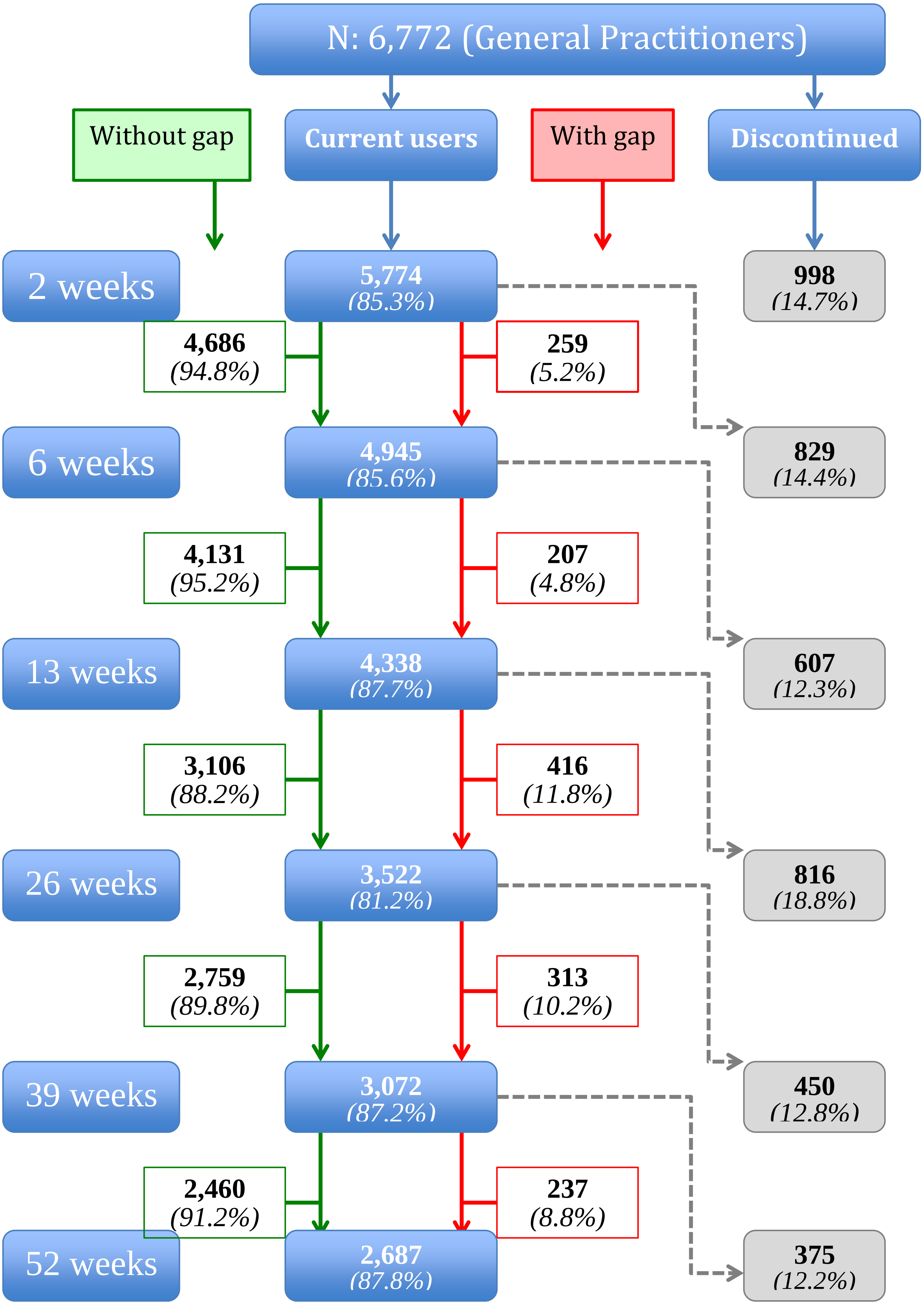

Supplement: Supplementary file 1 — Supplementary material 1 (PDF 446 kb) [file 345_2018_2557_MOESM1_ESM.pdf]
