# Supplementary material for: Continuation rates of alpha-blockers mono-therapy in adult men, prescribed by urologists or general practitioners: a pharmacy-based study
Source: World J Urol. 2018 Nov 13;37(8):1659–64. doi: 10.1007/s00345-018-2557-3 (PMC6684751; doi:10.1007/s00345-018-2557-3)

Figure 3. Numbers and percentages of patients who discontinued and continued α-blocker treatment (with or without gap) with first prescription by a urologist.

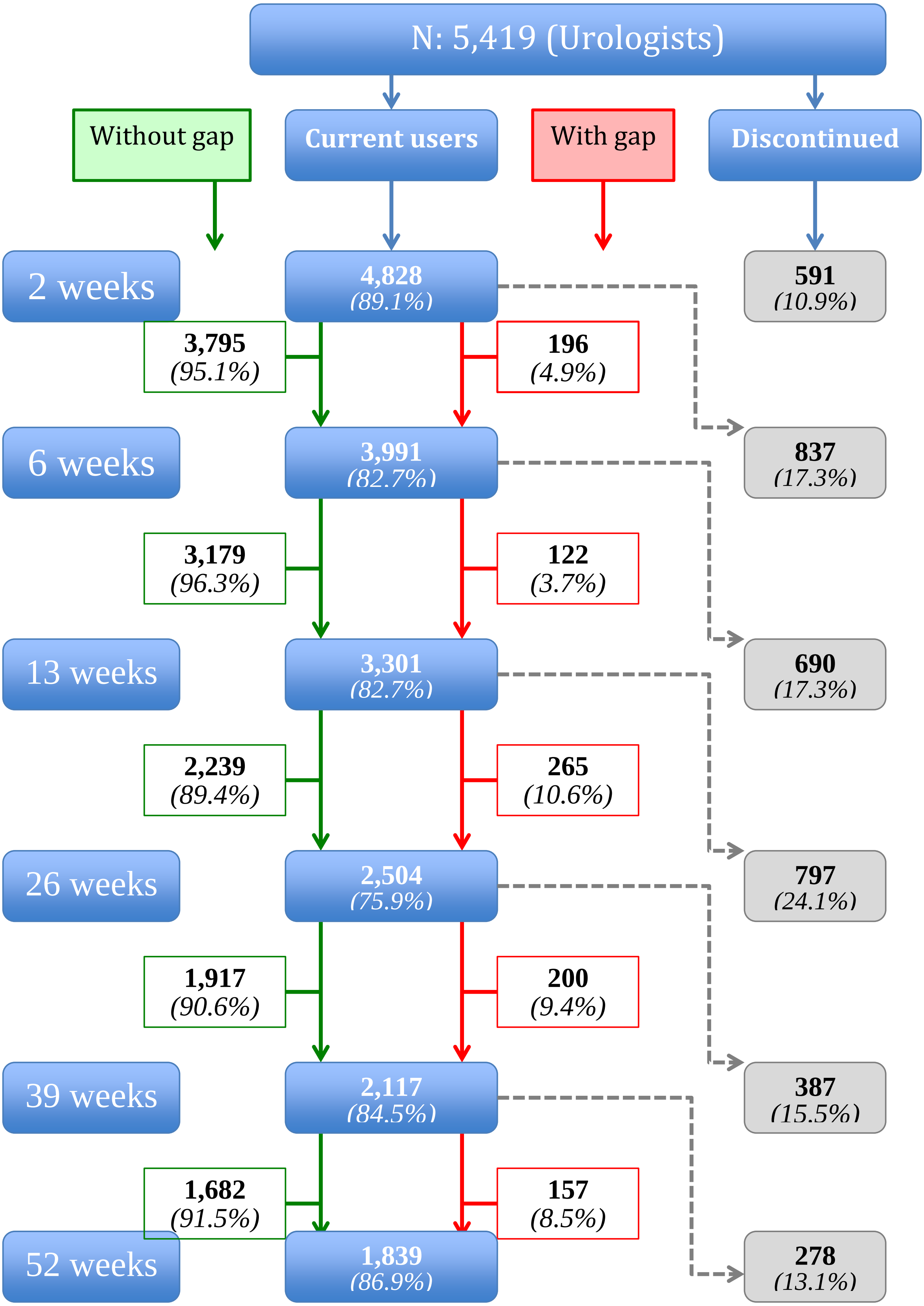

Supplement: Supplementary file 2 — Supplementary material 2 (PDF 443 kb) [file 345_2018_2557_MOESM2_ESM.pdf]
